# Readability of dermatology patient education materials: a comparative analysis of Turkish Dermatology Association brochures and texts generated by ChatGPT and Gemini

**DOI:** 10.55730/1300-0144.6342

**Published:** 2026-04-12

**Authors:** Ahmet Kağan ÖZDEMİR, Bengü Reyhan BOTSALI ÖZDEMİR, Akın AKTAŞ

**Affiliations:** 1 Department of Dermatology, Faculty of Medicine, Ankara Yıldırım Beyazıt University, Ankara, Turkiye; 2 Department of Dermatology, Ankara Etlik City Hospital, Ankara, Turkiye

**Keywords:** Health literacy, readability, artificial intelligence, patient education materials

## Abstract

**Background/aim:**

In modern healthcare, patient education materials (PEMs) are essential for bridging the gap between clinical knowledge and patient understanding. However, in Türkiye, where 64.6% of the adult population possesses inadequate or problematic health literacy, traditional medical brochures often exceed recommended reading levels. This study aimed to evaluate and compare the readability of Turkish dermatology PEMs prepared by the Turkish Dermatology Association (TDA) with those generated by artificial intelligence (AI) models.

**Materials and methods:**

A total of 68 TDA PEMs were analyzed. Readability was assessed using the Ateşman Readability Index. Responses were generated by AI models to questions extracted from these brochures under two conditions: “default” (no specific instructions) and “low-literacy prompted” (instruction to write at a sixth-grade level). Statistical comparisons were performed using the Kruskal–Wallis and Mann–Whitney U tests to evaluate differences in readability scores between the original TDA brochures and the four AI-generated groups. Effect size was calculated manually for each pairwise comparison and reported as r values.

**Results:**

The original TDA brochures had a mean readability score of 59.08 ± 5.80 (95% CI: 57.69–60.44), categorized as “medium difficulty.” All AI-generated groups demonstrated significantly higher readability scores compared with the TDA brochures (p < 0.05). Effect sizes for TDA versus AI group comparisons ranged from r = 0.242 to r = 0.753. In the default condition, Gemini Pro 2.5 produced more readable and consistent texts than ChatGPT-4.5 (66.63 versus 62.66; p = 0.004). While specific prompting significantly improved ChatGPT performance (p < 0.001), no significant change was observed in Gemini scores, which were already high in the default condition. Prompted Gemini achieved the highest overall mean score (68.98 ± 4.66).

**Conclusion:**

The linguistic complexity of TDA brochures may create a barrier for patients with limited health literacy. AI models, particularly when prompted with specific instructions, can generate dermatological PEMs with significantly higher readability levels.

## 1. Introduction

In modern healthcare, priority is placed on ensuring that patients fully comprehend the nature of their condition and available treatment options. However, relying solely on verbal communication is often insufficient, as patients tend to forget up to 80% of the medical information provided by healthcare practitioners [1]. In this context, patient education materials (PEMs) serve as an important tool to bridge the gap between clinical knowledge and patient understanding, thereby potentially increasing information retention. PEMs enhance patient self-efficacy and support disease self-management, while also improving comprehension and positively influencing health behaviors [2]. Moreover, patients demonstrate high engagement with these resources; approximately 77% apply the message and 87% remember the information presented in PEMs [3]. However, the actual value of these materials depends on whether patients can read and fully understand the information provided [2]. Current recommendations suggest that health education materials should be written at or below a fourth- to sixth-grade reading level [4,5]. Despite this, existing literature indicates that written health materials in dermatology often exceed the recommended reading level, which may negatively affect patient adherence and compliance [6,7].

Health literacy is defined as the ability to access, understand, appraise, and use information and services in ways that promote and maintain good health and well-being [8]. In Türkiye, the general health literacy index is reported as 30.4, which is significantly lower than the European average of 33.8 [8,9]. Categorical analysis indicates that 64.6% of the adult population falls into the “inadequate” or “problematic” categories, corresponding to approximately 35 million adults [9]. This limitation represents a barrier to effective communication in the Turkish healthcare system.

The challenge of creating readable materials is further complicated by the linguistic characteristics of the Turkish language and the specific nature of dermatology. Dermatology involves complex medical terminology and descriptive nomenclature that can be difficult to simplify. Moreover, as Turkish is an agglutinative language in which suffixes are added to root words to construct meaning, standard word lengths tend to be longer than those in Germanic languages. Consequently, readability metrics developed for English often fail to accurately assess Turkish texts, necessitating the use of language-specific tools such as the Ateşman Index, which account for these structural nuances without relying solely on translation-based formulas [10,11].

In addressing these linguistic and terminological challenges, artificial intelligence (AI)–based large language models (LLMs), such as ChatGPT and Gemini, have emerged as promising tools. Recent studies suggest that AI-generated PEMs can improve readability, providing a potentially effective strategy for presenting medical information to populations with limited health literacy [12,13]. However, it remains to be determined whether these AI models can overcome the structural characteristics of the Turkish language to provide health information that is easy for the average patient to read.

There is growing literature on LLM-generated PEMs, most of which focus on English, Spanish, or Chinese [14–17]. Comparative data evaluating the readability of AI-generated materials relative to traditional professional society brochures are limited, particularly in the field of dermatology in Türkiye. It is hypothesized that LLMs, particularly when prompted with specific grade-level instructions, will generate texts with significantly higher readability scores than standard brochures prepared by professional societies.

This study aimed to evaluate the readability of Turkish patient education materials in dermatology. The study also compared classic brochures prepared by the Turkish Dermatology Association (TDA) with Turkish patient information texts generated by ChatGPT and Google Gemini. This comparison was conducted to assess whether these AI tools can serve as facilitators for the Turkish population, which may struggle with the linguistic complexity of traditional health materials in dermatology.

## 2. Materials and methods

### 2.1. Study design and data collection

This study was conducted in two stages. Data collection and analysis were performed on 15 September 2025. In the first stage, all patient education brochures published by the TDA were obtained from the society’s official website.^1^ Before applying readability metrics, all noncontent items, including image captions, contact information, and references, were removed. Specifically, headings containing source questions were retained as sentences, whereas bulleted and numbered lists were removed, and their content was converted into individual sentences and integrated into continuous prose. This preprocessing was applied uniformly to both the original TDA brochures and all AI-generated texts. Representative examples of the original TDA brochure texts and the corresponding AI-generated outputs are provided in Appendix 1.

### 2.2. Readability analysis

Each text was evaluated using the Ateşman Turkish Readability Index, a tool adapted from the Flesch Reading Ease Index for the agglutinative structure of the Turkish language [10]. The Ateşman Index assesses readability based on two linguistic parameters: average word length (number of syllables per word) and average sentence length (number of words per sentence). Higher scores indicate easier readability. For each text, word and sentence counts were obtained using Microsoft Word (Microsoft Corp., Redmond, WA, USA). Syllable counts were determined based on the phonological structure of Turkish, in which each vowel corresponds to one syllable. Readability scores were subsequently computed in Microsoft Excel (Microsoft Corp., Redmond, WA, USA) using the Ateşman formula: readability score = 198.825 – (40.175 × average word length) – (2.610 × average sentence length), where average word length is defined as the number of syllables per word and average sentence length as the number of words per sentence [10,11]. The classification of readability scores according to the Ateşman Index is presented in Table [10,11].

### 2.3. AI-generated text production

All specific questions contained within each TDA brochure (e.g., “What are the symptoms?” and “How is it treated?”) were extracted to serve as inputs for the AI models. These questions were submitted individually to two large language models: ChatGPT-4.5 (OpenAI, San Francisco, CA, USA) and Gemini Pro 2.5 (Google LLC, Mountain View, CA, USA). For each model, responses were obtained under two separate conditions:

1. Default interaction: Each extracted question was submitted without any additional prompts or specific instructions.
2. Low-literacy interaction: A separate interaction was initiated for each question using a standardized prompt designed to elicit a response at a sixth-grade reading level. The specific prompt used was: “Answer the following questions in simple and clear language that a sixth-grade student can easily understand.”

Although the Ateşman Index does not directly correspond to grade-level metrics, the sixth-grade instruction was selected in alignment with international recommendations for PEM readability and has been used in comparable studies [4,5]. The prompt was intended to guide the model toward simplified language, and the Ateşman Index was used to quantitatively assess whether this simplification was achieved.

Both models were accessed exclusively through their official web interfaces under each platform’s default settings. No system prompts, fine-tuning, or modification of generation parameters were applied. Each question was submitted in a zero-shot manner, meaning that no examples or demonstrations were provided to the model, within a newly initiated session to ensure that prior interactions did not influence subsequent outputs. No output regeneration, filtering, or manual editing was performed. A single response per question per condition was recorded. Web browsing and retrieval-augmented generation features were disabled where applicable. The total number of questions extracted varied across brochures depending on the structure of the original material. All AI interactions were performed on 15 September 2025, using ChatGPT-4.5 (OpenAI San Francisco, CA, USA) and Gemini Pro 2.5 (Google LLC, Mountain View, CA, USA). Full prompts and representative sample outputs are provided in Appendix 1.

The responses generated under both conditions were recorded. All texts were converted into plain prose prior to readability analysis to avoid the aforementioned effects. All AI-generated texts were subsequently evaluated using the Ateşman Index in the same manner as the original brochures to allow direct comparison across groups.

### 2.4. Statistical analysis

All statistical analyses were performed using SPSS version 25.0 (IBM Corp., Armonk, NY, USA). Descriptive statistics for quantitative variables were presented as mean ± standard deviation (SD) and median (minimum–maximum). The normality of the data distribution was assessed using the Shapiro–Wilk test. Because the assumptions of normal distribution were not met, comparisons among the groups were conducted using the Kruskal–Wallis H test. When the Kruskal–Wallis test indicated a statistically significant difference, pairwise comparisons were performed using the Mann–Whitney U test with Bonferroni correction. Effect size was calculated manually for each pairwise comparison and reported as r values. Additionally, 95% confidence intervals for group means were estimated using bootstrap resampling (10,000 iterations). A p < 0.05 was considered statistically significant.

## 3. Results

This study evaluated the readability profiles of 68 patient education brochures published by the TDA, alongside their AI-generated counterparts across four experimental conditions. Within the original TDA texts, 53 brochures (77.9%) contained visual elements; consistent with the study protocol, these graphical components were excluded to ensure a strictly text-based readability assessment. The analysis of the original TDA brochures revealed notable variability in linguistic complexity. Readability scores within this group fluctuated substantially, ranging from a minimum score of 48.0, indicating “difficult” text, to a maximum of 70.3, corresponding to “easy” reading difficulty. The mean readability score for the original brochures was 59.08 ± 5.80, placing the material in the “medium difficulty” category according to the Ateşman Index classification.

**Figure 1:**
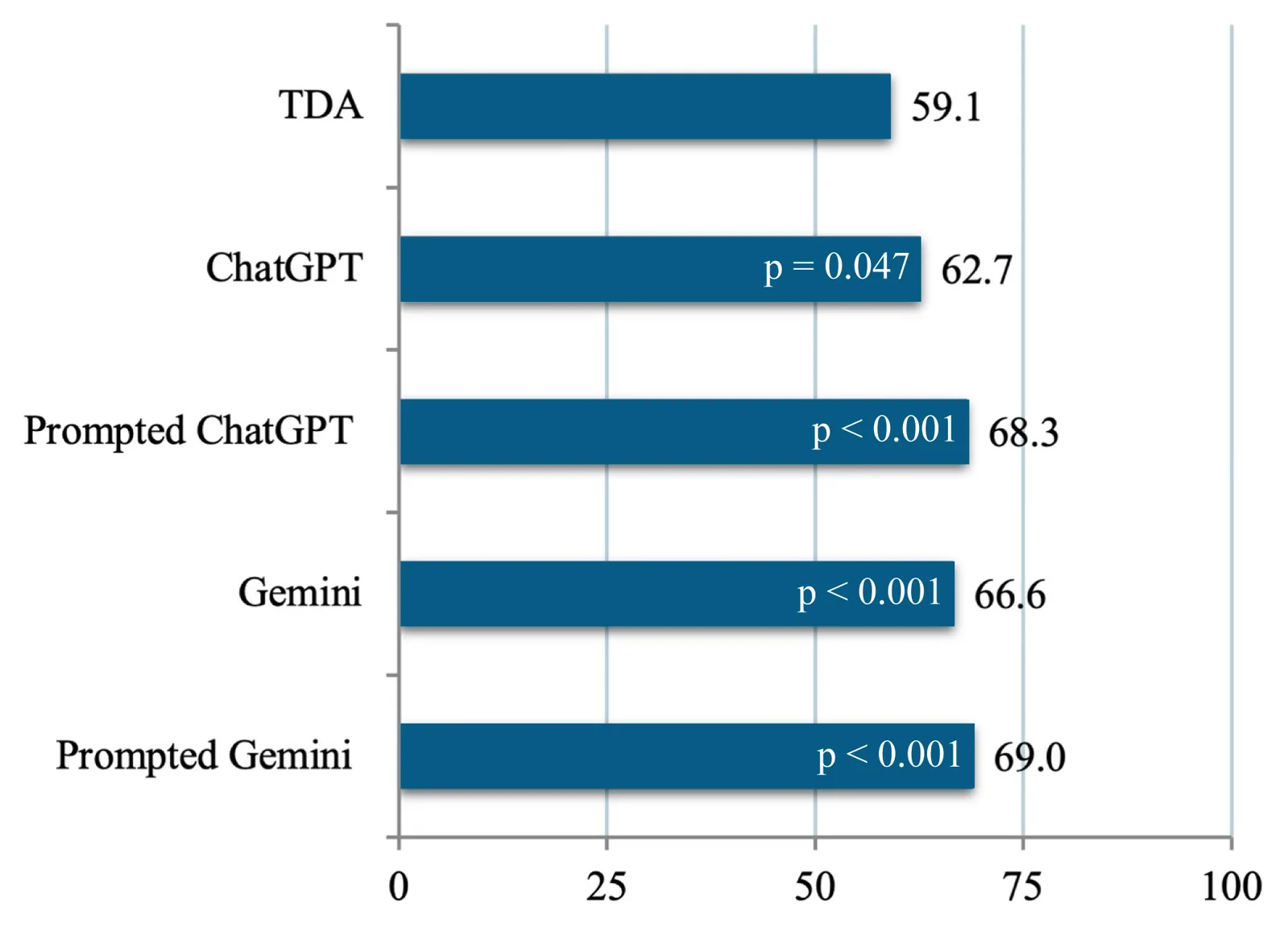
Mean Ateşman Readability Index scores across professional and artificial intelligence–generated patient education materials. A comparative analysis of the mean readability scores of the original TDA brochures and texts generated by ChatGPT-4.5 and Gemini Pro 2.5 under both default and low-literacy conditions. The p values indicated within the bars denote the level of statistical significance relative to the baseline TDA group. TDA: Turkish Dermatology Association.

A statistically significant difference was observed among the five study groups in terms of readability scores (p < 0.001). As illustrated in Figure 1, AI-generated texts consistently demonstrated higher readability levels than the original TDA texts. The mean readability scores (± SD) with 95% bootstrap confidence intervals were as follows: TDA, 59.08 ± 5.80 (95% CI: 57.69–60.44); ChatGPT, 62.66 ± 7.04 (95% CI: 61.03–64.37); prompted ChatGPT, 68.31 ± 5.39 (95% CI: 67.06–69.63); Gemini, 66.63 ± 4.03 (95% CI: 65.70–67.59); and prompted Gemini, 68.98 ± 4.66 (95% CI: 67.87–70.05). The prompted Gemini group achieved the highest overall performance, elevating the content to the threshold of the “easy” readability category. Pairwise comparisons confirmed that all AI-generated texts, regardless of the specific model or prompting strategy employed, scored significantly higher than the standard TDA brochures: TDA versus ChatGPT (p = 0.047; r = 0.242), TDA versus prompted ChatGPT (p < 0.001; r = 0.667), TDA versus Gemini (p < 0.001; r = 0.548), and TDA versus prompted Gemini (p < 0.001; r = 0.753).

**Figure 2:**
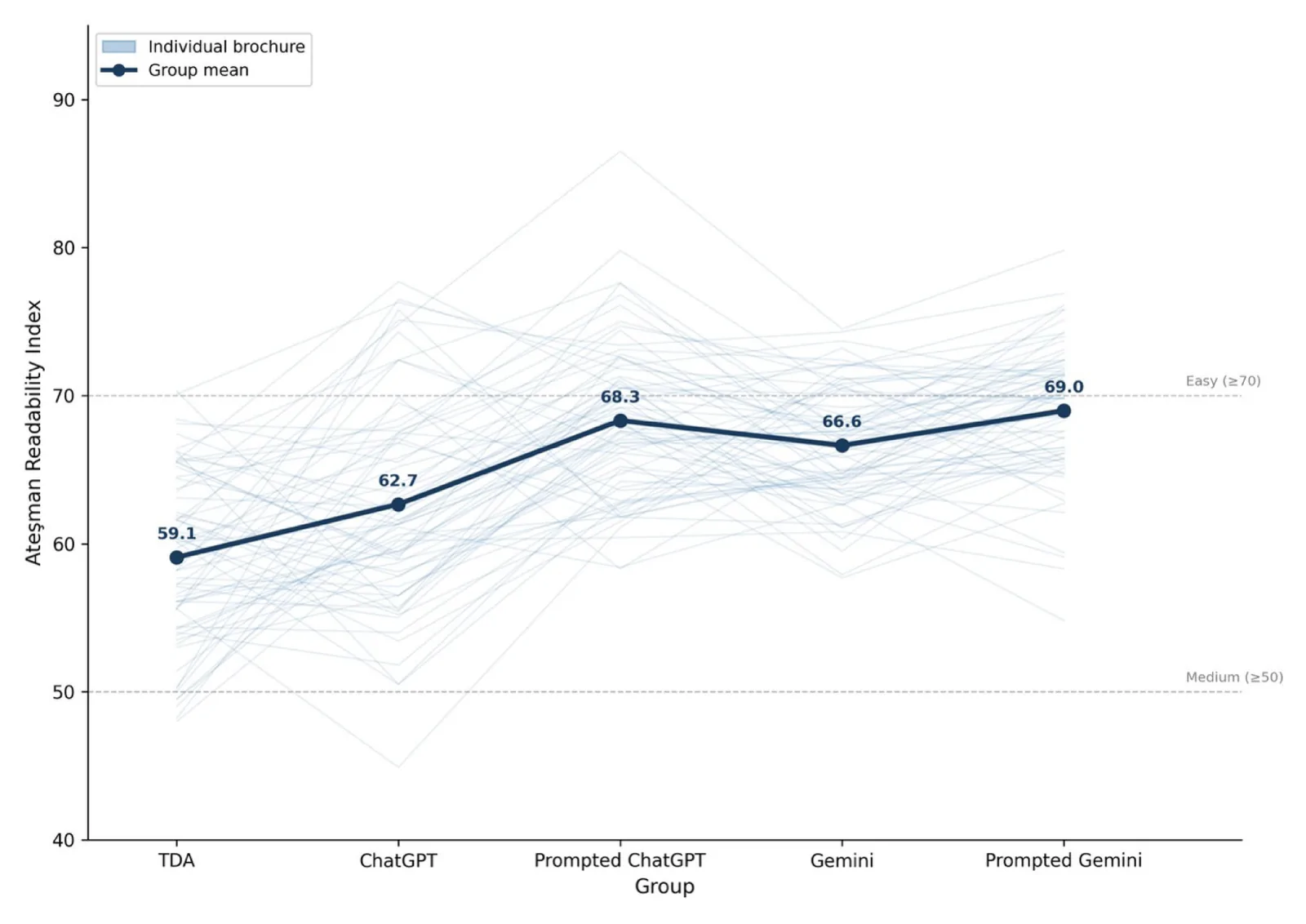
A spaghetti plot showing individual Ateşman Readability Index scores for each of the 68 TDA brochures across the five study groups. Each line represents a single brochure. The bold line indicates the group mean. Dashed reference lines denote the boundaries between Ateşman Readability Index categories. TDA: Turkish Dermatology Association.

A detailed examination of the models in their default interaction conditions revealed distinct patterns in text generation. Gemini produced significantly more readable texts than ChatGPT in the absence of specific prompts (66.63 versus 62.66; p = 0.004; r = 0.306). Furthermore, Gemini demonstrated greater consistency in its readability scores, with the lowest standard deviation (±4.03) among all study groups. This finding suggests that Gemini may have a greater capacity to simplify texts consistently, regardless of the original topic’s complexity, resulting in more stable outputs. In contrast, the default ChatGPT model exhibited the highest variability among all groups (±7.04), indicating that although it generally improved readability, its performance was less consistent without specific guidance. The variability in individual brochure scores across all five groups is illustrated in Figure 2.

The application of a standardized low-literacy prompt yielded different outcomes for the two models. For ChatGPT, the inclusion of the prompt significantly increased the mean score to 68.31 (p < 0.001; r = 0.425) and reduced baseline variability. Conversely, for Gemini, the addition of the prompt did not result in a statistically significant improvement over its default performance (p = 0.169). Lastly, when both models were optimized with specific prompts, no significant difference was observed between ChatGPT and Gemini (p = 1.000), indicating that both systems achieved comparable readability scores when instructed accordingly. Among the remaining pairwise comparisons, ChatGPT versus prompted Gemini also reached statistical significance (p < 0.001; r = 0.510).

## 4. Discussion

The results of this study indicate that traditional dermatology PEMs in Türkiye exhibit a level of linguistic complexity that may not align with the literacy needs of the general population. Specifically, the mean Ateşman Index score of 59.08 for the TDA brochures falls within the “medium difficulty” category, although it is closer to the lower boundary. When these findings are contextualized with national data indicating that 64.6% of the Turkish adult population possesses inadequate or problematic health literacy, it may be inferred that standard professional resources present a potential linguistic barrier, as reflected by readability metrics [9,18].

A central finding of this study is the significant improvement in readability scores achieved by both AI models. Gemini Pro 2.5 and ChatGPT-4.5 increased the readability levels of the materials. Gemini Pro 2.5 demonstrated greater consistency and higher readability scores in the default condition compared with ChatGPT-4.5, which exhibited higher variability. This may suggest that Gemini performs more consistently in generating readable Turkish texts; however, this observation requires further investigation. Furthermore, the absence of a significant difference between the two Gemini conditions may indicate a potential ceiling effect, whereby the default outputs reached relatively high readability scores, leaving limited room for further improvement through additional prompting. This observation may imply that further simplification beyond a certain level could risk altering the medical meaning of the text; however, this interpretation should be considered with caution.

The improvement in ChatGPT-4.5 performance following the use of a low-literacy prompt highlights an important consideration in the clinical application of AI for patient education. LLMs can generate dermatologic patient education materials at targeted reading levels when appropriately prompted, as supported by studies demonstrating improved readability in dermatology contexts across various languages [17,19]. A recent tutorial on prompt engineering emphasizes that designing effective prompts is an emerging skill for health care professionals, as the clarity, specificity, and contextual framing of instructions substantially influence how LLMs interpret tasks and generate clinically relevant outputs [20]. This includes explicitly defining the intended audience and educational objectives to guide models toward usable and appropriate responses in patient education settings [20]. Collectively, these findings suggest that literacy-oriented prompting may represent a practical competency for clinicians aiming to integrate LLMs into patient education while aligning outputs with health literacy standards.

The initial findings on the readability of TDA PEMs reflect a broader global challenge in dermatology. Research focusing on major American dermatology organizations has shown that their patient materials consistently exceed recommended reading levels [7]. Similar obstacles have been identified in the United Kingdom, where leaflets from the British Association of Dermatologists often present significant readability barriers [6]. This issue appears to be widespread across dermatologic subspecialties, as observed in pediatric dermatology, where linguistic barriers may limit the effective delivery of care to families [17,21].

The present study contributes to the growing body of evidence supporting the use of AI in non-English healthcare settings. Recent evidence suggests that AI tools may outperform traditional approaches in translating patient handouts, providing more readable and contextually appropriate results across different languages [22]. While the reliability of AI responses may vary across languages, these models have demonstrated the ability to provide relatively consistent and relevant health information across multiple language contexts [18].

The quantitative improvement in readability scores is further contextualized by evidence regarding patient and clinician preferences. Research has shown that both patients and physicians may prefer AI-generated information over materials provided by professional societies [23,24]. However, while patients may rate AI outputs as comparable in terms of comprehensiveness and readability, physicians have reported that these outputs may be clinically inferior [12,25,26]. This discrepancy highlights the need to reconcile patient-preferred accessibility with professional standards of clinical care.

In contrast to the present findings, conflicting results have been reported in the literature regarding the effect of LLMs on dermatologic PEM readability. For example, an analysis of four common (atopic dermatitis, acne vulgaris, herpes zoster, and psoriasis) and four rare conditions (epidermolysis bullosa, lichen planus, bullous pemphigoid, and lamellar ichthyosis) revealed that American Academy of Dermatology PEMs exhibit low readability levels [19]. Notably, LLMs produced more complex content when used without specific prompts, consistently exceeding the readability levels of the original materials. However, consistent with the present findings, the use of targeted prompts in that study increased the readability of the aforementioned materials [19]. Another study by Gawey et al., focusing on hidradenitis suppurativa, found that ChatGPT produced content that was more complex than materials from professional disease foundations and performed worse than general health websites [27]. Further reinforcing these concerns, research by Verran showed that an unprompted AI model, when tested with common diseases and treatments included in British Association of Dermatologists’ patient information leaflets, generated materials that often exceeded target readability thresholds [15]. Beyond linguistic difficulty, the study also reported a gap in clinical depth, as the AI did not address critical patient concerns such as curability and heritability [15].

Several limitations should be acknowledged. The Ateşman Index is a quantitative metric and does not evaluate qualitative aspects such as tone, visual layout, or content completeness. Furthermore, visual elements were excluded from the analysis, as only textual content is evaluated by the Ateşman Index. Given that visuals constitute a critical component of PEMs, particularly in dermatology, overall educational effectiveness should not be inferred from these findings. It should be noted that readability does not necessarily equate to comprehensibility from the reader’s perspective, as factors such as prior knowledge, health literacy level, and contextual understanding influence how written information is processed. Importantly, clinical accuracy and completeness are not implied by higher readability scores. Simplified language may be achieved by AI models through the omission of clinically important caveats, warnings, or disease-specific details. No expert panel reviewed the content for medical accuracy; therefore, the findings should be interpreted strictly as a linguistic evaluation. The analysis was restricted to formal brochures available on the TDA website, and other formats, such as news updates or video transcripts, were excluded. Additionally, although different LLMs were evaluated, the specific impact of various versions or iterative updates within the same model was not assessed.

## 5. Conclusion

In conclusion, although professional society brochures are considered the gold standard for clinical accuracy, their linguistic complexity may constitute a significant barrier for a large proportion of the Turkish population with limited health literacy. This study indicates that LLMs such as ChatGPT and Gemini can generate dermatological PEMs with significantly higher readability scores. It should be noted that the use of specific, readability-focused instructions appears to be an important factor in achieving consistent results across different AI platforms. A hybrid approach, in which AI is used to simplify evidence-based content from professional societies, may represent a viable strategy for producing more readable patient education materials in dermatology.

## Figures and Tables

**Table:** Classification of the Ateşman Readability Index.

Score	Difficulty level
90–100	Very easy
70–89	Easy
50–69	Medium
30–49	Difficult
0–29	Very difficult

## Data Availability

The data supporting the findings of this study are available from the corresponding author upon reasonable request.

## Footnotes

1 Turkish Dermatology Association (2025). Hasta Bilgilendirme Broşürleri ve Kitapları (in Turkish) [online]. Website https://turkdermatoloji.org.tr/icerik/detay/210 [accessed 15 September 2025].

## Supplementary file

### Appendix 1A AI text generation protocol

Both models (ChatGPT-4.5, OpenAI; Gemini Pro 2.5, Google) were accessed through their official web interfaces under default settings. No system prompts, fine-tuning, or modification of generation parameters were applied.

For each of the 68 TDA brochures, all questions contained within the brochure were extracted manually and submitted individually to each model under two conditions:

1. Default condition: Each question was submitted as-is, without any additional instructions.
2. Low-literacy prompted condition: A separate interaction was initiated for each question, utilizing the following standardized prompt:

*“Answer the following questions in simple and clear language that a 6th-grade student can easily understand.”*

Each question was submitted in a zero-shot manner within a newly initiated session. No output regeneration, filtering, or manual editing was performed. A single response per question per condition was recorded. Web browsing and retrieval-augmented generation features were disabled where applicable. The total number of questions varied across brochures depending on the structure of the original material. All interactions were performed on September 15, 2025.

### Appendix 1B Representative sample outputs

The following sections present the texts for the Morfea brochure as a representative example. All texts are in Turkish, as the study evaluates Turkish-language patient education materials. Texts are shown in their plain-prose format as analyzed; bulleted and numbered lists were converted into individual sentences and integrated into continuous prose prior to readability scoring, as described in subsection 2.1 of the manuscript.

The eight questions extracted from the TDA Morfea brochure and submitted to each model under both conditions are listed below in Turkish:

1. Morfea kimlerde, ne sıklıkta, ne zaman görülür?
2. Morfeanın nedeni nedir?
3. Morfea’nın belirtileri nelerdir?
4. Hastalık ilerleyici midir?
5. Morfea’nın yarattığı sorunlar nelerdir?
6. Morfea tanısı nasıl konur?
7. Morfea’nın seyri nasıldır?
8. Morfea nasıl tedavi edilir?

#### 1B.1 TDA original text

The original TDA text in Turkish was obtained from the Turkish Dermatology Association official website^1^, and noncontent elements, including image captions, contact information, and references, were removed prior to analysis.

#### Morfea kimlerde, ne sıklıkta, ne zaman görülür?

Morfea oldukça nadir görülen bir hastalıktır. Hastalık kadınlarda 2–3 kat daha fazla görülür. Ortalama hastalık başlangıcı 40’lı yaşlardır.

#### Morfeanın nedeni nedir?

Hastalığın nedeni tam olarak aydınlatılamamıştır. Travma, radyasyon, ilaçlar, infeksiyonlar, genetik ve otoimmünite üzerinde durulmaktadır. Nedeni ne olursa olsun, hastalık süreci damarsal hasar ve deride elastikiyeti sağlayan bir protein olan kolajen üretiminin artışı ve yıkımının azalmasıyla ilerler.

#### Morfea’nın belirtileri nelerdir?

Morfea sıklıkla yalnızca deriyi etkiler. Bazı olgularda ise etkilediği deri alanının altındaki yağ, kas vb. dokular da etkilenebilir. Sıklıkla gövde ön veya arka yüzde yerleşirlerse de, yüz dahil tüm deri alanları etkilenebilir. Aktif lezyonlar, sıklıkla 2–15 cm büyüklüğünde, merkezinde pigment değişiklikleri olan pembemsi mor, kenarları leylak renginde oval yamalardır.

#### Hastalık ilerleyici midir?

Zamanla daha açık tonda görünen merkezi kısım giderek sertleşir, kalınlaşır, kuru ve parlak bir görünüm kazanır. Etkilenen alanlarda zaman içerisinde kıllar ve ter bezlerinde de kayıplar gelişebilir. Kollar ve bacaklarda yerleştiklerinde doğrusal özellik kazanabilir. Deri altı yapıları tutarak hareket kısıtlılıklarına yol açabilir. Yüzü tutan şiddetli bir formunda, kas ve alttaki kemik yapılar da etkilenerek önemli görünüm sorunlarına yol açabilir.

#### Morfea’nın yarattığı sorunlar nelerdir?

Görünür alanlarda yerleştiğinde kozmetik görünüm nedeniyle özgüven sorunları, kollar ve bacaklarda hareket kısıtlılıkları, yaygın ve çok sayıda bulgu ile seyredenlerde deride yaygın sertleşmeler ortaya çıkabilir.

#### Morfea tanısı nasıl konur?

Muayene ve gerektiğinde bir deri biyopsisi tanıda yardımcıdır. Yaygın ve doğrusal formlarda bazı bozukluklara rastlanabilirse de, kan testleri sıklıkla normaldir.

#### Morfea’nın seyri nasıldır?

Morfea, sıklıkla yıllarca devam edip ardından gerileme gösterir. Kollar ve bacaklarda yerleşen doğrusal formlar sıklıkla kalıcı değişiklikler oluşturup dirençli seyredebilir. Özellikle yüzde yerleşenlerde nöroloji veya göz muayeneleri yaptırılmalıdır.

#### Morfea nasıl tedavi edilir?

Morfea sıklıkla kendiliğinden yıllar içerisinde gerileme gösterir. Etkilenen alanlarda renk ve kıvama ilişkin bazı değişiklikler kalıcı olabilir. Doğrusal formlar daha dirençlidir. Hastalığın erken dönemlerinde daha etkili olmak üzere, aşağıdaki tedaviler uygulanabilmektedir. Tedaviler: (A) Sınırlı alanlar için: kortizonlu krem ve merhemler, D vitamini benzeri kremler (Kalsipotriol), bağışıklık sistemini düzenleyici kremler ve merhemler (Takrolimus/Pimekrolimus). (B) Yaygın deri alanları etkilenmiş veya şiddetli formlar mevcutsa: sistemik kortizon içeren tabletler, metotreksat, UVA-1 veya dar band UVB fototerapisi.

#### 1B.2 ChatGPT-4.5 — default condition

Model: ChatGPT-4.5 (OpenAI). No additional instructions were provided. Each question was submitted individually in a zero-shot session.

#### Morfea kimlerde, ne sıklıkta, ne zaman görülür?

Her yaşta ortaya çıkabilir, ancak genellikle yetişkinlerde ve kadınlarda daha sık görülür. Hastalığın görülme sıklığı nadirdir.

#### Morfeanın nedeni nedir?

Nedeni tam olarak bilinmemekle birlikte, bağışıklık sisteminin anormal yanıtı, genetik ve çevresel faktörlerin rol oynadığı düşünülmektedir.

#### Morfea’nın belirtileri nelerdir?

Ciltte başlangıçta pembe veya mor renkte, sınırları belirgin lekeler oluşur. Zamanla bu alanlar sertleşir, kalınlaşır ve bazen renk değişiklikleri görülür.

#### Hastalık ilerleyici midir?

Morfea genellikle yavaş ilerler ve sınırlı bir alanda kalır, ancak bazı durumlarda daha geniş alanları etkileyebilir.

#### Morfea’nın yarattığı sorunlar nelerdir?

Deride sertleşme ve kalınlaşmaya bağlı hareket kısıtlılığı, estetik sorunlar ve nadiren iç organ tutulumları olabilir.

#### Morfea tanısı nasıl konur?

Klinik muayene ve deri biyopsisi ile tanı konulur.

#### Morfea’nın seyri nasıldır?

Hastalık genellikle birkaç yıl süren aktivite dönemi sonrası stabil veya gerileyici bir seyir gösterebilir.

#### Morfea nasıl tedavi edilir?

Tedavi topikal kortikosteroidler, kalsinörin inhibitörleri, fototerapi ve sistemik immünmodülatör ilaçlar ile yapılabilir. Tedavi hastalığın yaygınlığına ve şiddetine göre değişir.

#### 1B.3 ChatGPT-4.5 — low-literacy prompted condition

Model: ChatGPT-4.5 (OpenAI). A separate interaction was initiated for each question using the standardized prompt described in Appendix 1A.

#### Morfea kimlerde, ne sıklıkta, ne zaman görülür?

Morfea hem çocuklarda hem de yetişkinlerde görülebilir, ancak kadınlarda biraz daha sık rastlanır. Genellikle genç yaşlarda başlar ama her yaşta ortaya çıkabilir. Nadir görülen bir hastalıktır.

#### Morfeanın nedeni nedir?

Hastalığın nedeni tam olarak bilinmemektedir. Bağışıklık sisteminin cilde karşı aşırı tepki vermesiyle ortaya çıkar. Bazı enfeksiyonlar, travmalar veya çevresel etkenler tetikleyici olabilir.

#### Morfea’nın belirtileri nelerdir?

Deride sert, parlak ve genellikle mor ya da kahverengi renkli lekeler; bu alanlarda saç dökülmesi ve terlemenin azalması; ciltte incelme veya çekilmeler; nadiren eklem kısıtlılığı görülebilir.

#### Hastalık ilerleyici midir?

Morfea genellikle yavaş ilerler. Bazı durumlarda sınırlı kalırken, bazen çevre dokulara yayılabilir. Organlara zarar vermez.

#### Morfea’nın yarattığı sorunlar nelerdir?

Ciltte sertlik ve şekil bozukluğu, hareket kısıtlılığı, görsel rahatsızlık; çocuklarda büyüme çağında uzuv gelişiminde farklılık olabilir.

#### Morfea tanısı nasıl konur?

Tanı genellikle cilt muayenesiyle konur. Gerekirse cilt biyopsisi yapılabilir. Bazı durumlarda kan testleri veya görüntüleme yöntemleri de kullanılır.

#### Morfea’nın seyri nasıldır?

Bazı morfea tipleri birkaç yıl içinde kendiliğinden durabilir. Ancak bazı tiplerde ciltte kalıcı izler kalabilir. Düzenli takip gerektirir.

#### Morfea nasıl tedavi edilir?

Kortizonlu kremler veya haplar, bağışıklık sistemini düzenleyici ilaçlar, fizik tedavi (özellikle eklem etkilenmişse), cilt bakım kremleri kullanılabilir. Tedavi, hastalığın yaygınlığına ve şiddetine göre planlanır.

#### 1B.4 Gemini Pro 2.5 — default condition

Model: Gemini Pro 2.5 (Google). No additional instructions were provided. Each question was submitted individually in a zero-shot session.

#### Morfea kimlerde, ne sıklıkta, ne zaman görülür?

Morfea, genel olarak nadir görülen bir hastalıktır. En sık olarak çocukluk çağında (2–14 yaş arası) ve orta yaşlı yetişkinlerde (20–50 yaş arası) ortaya çıkar. Kadınlarda erkeklere göre yaklaşık 3 kat daha sık görülür.

#### Morfeanın nedeni nedir?

Hastalığın nedeni tam olarak bilinmemektedir, ancak otoimmün bir hastalık olduğu düşünülmektedir. Genetik yatkınlık, Lyme hastalığı gibi enfeksiyonlar veya travma gibi faktörlerin hastalığı tetikleyebileceği düşünülmektedir.

#### Morfea’nın belirtileri nelerdir?

Morfea başlangıçta kırmızımsı veya morumsu bir leke olarak ortaya çıkar. Zamanla bu leke merkezden dışa doğru genişleyerek sertleşir ve parlak bir görünüm alır. Lezyonlar tipik olarak ‘leylak halkası’ olarak bilinen bir görünüme sahip olabilir.

#### Hastalık ilerleyici midir?

Morfea genellikle kendi kendini sınırlayan bir hastalıktır ve birkaç yıl içinde kendiliğinden inaktif hale gelebilir. Ancak ciltte kalıcı hasar ve izler kalabilir. Özellikle lineer morfea, alttaki kas ve kemikleri etkileyerek uzuvlarda kısalığa ve deformiteye neden olabilir.

#### Morfea’nın yarattığı sorunlar nelerdir?

Kozmetik sorunlar (özellikle yüz, boyun veya el gibi açıkta kalan bölgelerde); hareket kısıtlılığı; çocukluk çağında başlayan lineer morfeada büyüme geriliği ve kas-kemik atrofisi görülebilir.

#### Morfea tanısı nasıl konur?

Tanı genellikle bir dermatolog tarafından fiziksel muayene ve hastanın öyküsü ile konulur. Kesin tanı için etkilenen ciltten biyopsi alınarak patolojik inceleme yapılır.

#### Morfea’nın seyri nasıldır?

Hastalığın seyri değişkendir. Çoğu hasta birkaç yıl içinde iyileşme evresine girer, ancak ciltte kalıcı renk değişiklikleri ve sertlik kalabilir.

#### Morfea nasıl tedavi edilir?

Topikal ilaçlar (kortizonlu kremler, D vitamini türevleri veya takrolimus); fototerapi (UVA1, dar bant UVB veya PUVA); sistemik ilaçlar (metotreksat, kortikosteroidler); fizik tedavi (eklem bölgelerini etkileyen vakalarda) kullanılabilir.

#### 1B.5 Gemini Pro 2.5 — low-literacy prompted condition

Model: Gemini Pro 2.5 (Google). A separate interaction was initiated for each question using the standardized prompt described in Appendix 1A.

#### Morfea kimlerde, ne sıklıkta, ne zaman görülür?

Morfea her yaşta ve cinsiyette görülebilse de en sık 20–50 yaş arasındaki kadınlarda görülür. Çocuklarda da görülebilir. Genellikle nadir görülen bir hastalıktır.

#### Morfeanın nedeni nedir?

Morfea’nın nedeni tam olarak bilinmiyor. Ancak vücudun bağışıklık sisteminin kendi dokularına saldırmasıyla ortaya çıkan bir hastalık olduğu düşünülüyor. Genetik yatkınlık ve bazı tetikleyici faktörler (yaralanma, böcek ısırığı gibi) hastalığın oluşmasında rol oynayabilir.

#### Morfea’nın belirtileri nelerdir?

En belirgin belirtisi ciltte oluşan sert, mumsu ve oval şekilli plaklardır. Bu plaklar ilk başta kırmızı veya mor renkte olabilir, zamanla ortası beyazlaşır ve kenarları mor bir halka ile çevrili hale gelir. Plaklar kaşıntı yapabilir ve ciltte gerginlik hissi yaratabilir.

#### Hastalık ilerleyici midir?

Morfea genellikle ilerleyici bir hastalık değildir. Plaklar zamanla kendiliğinden veya tedaviyle iyileşir. Ancak bazı nadir durumlarda hastalık daha geniş bir alana yayılabilir.

#### Morfea’nın yarattığı sorunlar nelerdir?

Morfea genellikle kozmetik bir sorun yaratır. Ciltte oluşan izler ve renk değişiklikleri rahatsız edici olabilir. Plaklar eklemlerin üzerinde oluşursa eklem hareketlerini kısıtlayabilir.

#### Morfea tanısı nasıl konur?

Tanı koymak için dermatolog sertleşmiş cilt plaklarını inceler. Kesin tanı için etkilenen bölgeden küçük bir deri parçası alınarak (biyopsi) incelenmesi gerekir.

#### Morfea’nın seyri nasıldır?

Morfea genellikle zamanla iyileşen bir hastalıktır. Plaklar aktif bir dönemden sonra pasif hale gelir ve yavaşça yumuşayarak düzelir. Bu süreç birkaç aydan birkaç yıla kadar sürebilir.

#### Morfea nasıl tedavi edilir?

Tedavide amaç hastalığın ilerlemesini durdurmak ve belirtileri hafifletmektir. Kremler ve merhemler, ışık tedavisi (fototerapi) ve ağızdan alınan ilaçlar kullanılabilir.
